# A comprehensive review of thermoelectric cooling technologies for enhanced thermal management in lithium-ion battery systems

**DOI:** 10.1016/j.heliyon.2024.e40649

**Published:** 2024-11-27

**Authors:** Mehwish Khan Mahek, Mohamad Ramadan, Sharul Sham bin Dol, Mohammed Ghazal, Mohammad Alkhedher

**Affiliations:** a Mechanical and Industrial Engineering Department, Abu Dhabi University, Abu Dhabi, 59911, United Arab Emirates; b School of Engineering, International University of Beirut BIU, Beirut, Lebanon; c School of Engineering, Lebanese International University LIU, Bekaa, Lebanon; d Electrical, Computer, and Biomedical Engineering Department, Abu Dhabi University, Abu Dhabi, United Arab Emirates

**Keywords:** Lithium-ion battery, Thermoelectric cooling, Hybrid BTMS, TEC-Based BTMS

## Abstract

With the rising demand of electric vehicles (EVs) and hybrid electric vehicles (HEVs), the necessity for efficient thermal management of Lithium-Ion Batteries (LIB) becomes more crucial. Over the past few years, thermoelectric coolers (TEC) have been increasingly used to cool LIBs effectively. This study provides a comprehensive analysis of thermoelectric technologies for improving the thermal management in LIB Systems. The review examines core ideas, experimental approaches, and new research discoveries to provide a thorough investigation. The inquiry starts with analysing TEC Hybrid battery thermal management system (BTMS) Cooling, including air cooled, phase change material (PCM)-cooled, liquid cooled, and heat pipe cooled thermoelectric BTMS. This paper also examines the shape, thickness, and arrangement of heat sink fins in TECs, providing valuable insights for enhancing thermal efficiency. The review additionally focuses on Control in TEC-based Li-ion BTMS, emphasizing the need for effective regulatory systems. Recognizing inherent limits, the study examines issues, stimulating future research possibilities. It encourages a complete grasp of contemporary thermoelectric technologies in Li-ion BTMS by synthesizing theoretical frameworks, experimental data, and literature assessments. The review also examines Future Directions, emphasizing prospects for innovation. A collaborative future is envisioned in which shared information drives long-term advances in energy storage technologies.

**Table:** Nomenclature

**Abbreviations**	
EV	Electric Vehicles
HEV	Hybrid Electric Vehicles
LIB	Lithium-Ion Batterie
TEC	Thermoelectric Coolers
BTMS	Battery Thermal Management System
PCM	Phase Change Material
TEG	Thermoelectric Generator
COP	Coefficient Of Performance
SOC	State Of Charge
TEM	Thermoelectric Module
DC	Direct Current
VFERI	Vehicle, Fuel, And Environments Research Institute
CPCM	Composite Phase Change Material
RTR	Rate Of Temperature Rise
NF	Nanofluid
PID	Proportional Integral Derivative
NMPC	Nonlinear Model Predictive Control
NN	Neural Networks
FOM	Figure Of Merit
DOS	Density Of States
AI	Artificial Intelligence

## 1 Introduction

Energy shortage and environmental degradation are two big global issues. The principal oil consumption sector, the car industry, is inextricably linked to these two challenges. Most automotive vehicles run on fossil fuels, and the quantity of fossil fuel available is diminishing by the day [1,2]. Consequently, there has been a swift surge in the need for renewable energy, resulting in its proportion in worldwide power production consistently ascending [3]. Despite continuous usage, the confirmed petroleum resource reserves have not increased significantly in the last five decades [4]. The automotive sector, reliant on fossil fuels, is encountering significant obstacles. EVs have garnered growing attention due to their capacity to improve vehicle economy, encourage environmentally conscious behaviours, and reduce dependence on oil. They are regarded as a prominent pathway for future automobile technology development [5,6]. As reported by BNEF, the global number of EVs have surpassed 26 million by the conclusion of 2022 [7]. This is a substantial growth in comparison to the 1 million EVs recorded in 2016. Global electric vehicle EV sales and LIB shipments are seeing a consistent increase [8]. The primary obstacle to the commercialization of EVs is in the energy storage domain. Creating a practical energy storage technology that can attain both high power and high energy is crucial. To meet EVs' power and energy needs, LIBs are coupled in series or parallel configurations to create module and pack structures [9,10].

LIBs offer significant advantages as the primary energy storage technology in EVs, including high power density, reduced emissions, no memory effect, along with a long cycle life [[11], [12], [13]]. LIBs are considered to be susceptible to pressure, vibration, and temperature in these challenging applications. Temperature significantly influences on the performance of LIBs [14,15]. Research investigating the use of LIB for high-power discharge has uncovered the risk of overheating, which may result in reduced battery capacity and lifespan [16,17]. In severe instances, excessive heat may lead to dangerous consequences such as the ignition or detonation of electrical components inside the vehicle [18]. EVs are less prevalent in warmer climates due to their increased susceptibility to overheating compared to traditional automobiles. There are two factors contributing to this phenomenon. One reason for the underperformance of batteries in electric cars during hot weather is the decrease in electron mobility caused by elevated temperatures, resulting in a reduction in available power. The second is that EVs dissipate heat independently of internal combustion engines. Furthermore, the absence of an internal combustion engine renders the battery incapable of dissipating heat efficiently, potentially resulting in overheating and a reduction in operational range. Research on the adoption of EVs is increasing, with a particular focus on settings in Northern Europe. While these theories and models contribute significantly to the comprehension of technology adoption and EV adoption in general, their applicability to EV adoption in Kuwait and the GCC is limited. At present, the GCC nations have a negligible impact on the adoption of alternative non-carbon fuels for ground transportation, as EVs comprise less than 1 % of the total number of ground vehicles. The reason behind this is because the GCC nations have a very hot environment, which significantly affects the usage and appropriateness of automobiles, in contrast to countries situated in Northern Europe [19,20]. Additionally, the performance of electric cars depends on critical aspects such as battery capacity, discharge rate, and operating temperature [21]. An examination of batteries revealed that the optimal working temperature must not exceed 60 °C. In particular, the recommended operating range of temperatures for LIBs is between 22 °C and 60 °C, with a temperature differential of 5 °C [22]. Enhancing temperature uniformity and maintaining the operational temperature of the battery within a suitable range are the principal objectives of BTMS in EVs. As a solution to this issue, various types of BTMS have been developed and evaluated by researchers and manufacturers [23].

The battery surface temperature must be assessed in conjunction with the internal temperature profile of the battery. To get a more precise simulation of BTMS, it is essential to construct heat-transfer models of the exterior cooling structures. In conjunction with the electrochemical and thermal models, many battery models are used to infer the internal state of battery cells [[24], [25], [26]]. Due to the challenge of directly detecting the temperature within battery cells, the internal temperature may be estimated by analyzing the temperature distribution on the battery surface [27]. Mahamud and Park [28] designed a thermal model with spatial resolution and lumped capacitance, enabling rapid prediction of cell temperature over various operational cycles.

Over the past few years, TEC have been employed to cool the batteries in EVs. When electricity is provided, TECs shift heat from the cold end of the material to the hot end. A significant temperature difference is created at the two ends of the semiconductor in this device by applying an electric current to semiconductor devices. After that, cooling may be accomplished using the cold end [29]. In the BTMS, the TEC's hot end is cooled by water, while the cold end is linked to the battery. To manage the heat accurately, the TEC acts as a heat pump between the cooling medium and the battery [30]. The reliability and manageability of TECs are widely established, and they may be readily managed to regulate the temperature of specific battery cells. This characteristic is beneficial for EV and HEV applications since local temperatures might vary considerably [31].

Applications for thermoelectric elements may be split into two distinct categories. One is the thermoelectric generator (TEG), which operates on the Seebeck effect. This device harnesses waste heat and converts it into electricity. The TEC, which employs the Peltier effect to transform electrical energy into thermal energy, serves as the alternative.

In order to maintain the working temperatures of LIBs in severe environments, Luo et al. [32] proposed a BTMS that features TEC, liquid cooling, and composite phase change material (CPCM). A transient numerical model was developed to provide a precise evaluation of the system's performance, which encompassed the multiphysics domains of thermal, electrical, and fluid. The maximal battery temperature and CPCM liquid percentage decreased as the mass fraction of expanded graphite (EG), the input current of the TEC cooling system, and the discharge rate of the coolant increased. The temperature difference between batteries decreased as the EG mass percentage increased, but it increased with current and coolant flow speed. The BTMS exhibits the most efficient cooling performance and the lowest power consumption when operating at a 12 % EG mass fraction, 3 A TEC cooling input current, and 0.05 m/s refrigerant flow rate. The battery pack can be heated to 293.15 K from 263.15 K in 5600 s and 2240 s, respectively, by TEC preheating input currents of 4 A and 5 A. Zhao et al. [33] investigated a TEC system that utilizes PCM heat storage for the purpose of cooling in space applications and discovered that it is possible to enhance the cooling power. Liu et al. [34] used TEC to examine the thermal management system for a prismatic LIB pack. A substantial temperature differential may result in the pack being cooled at a high ambient temperature, surpassing the capabilities of natural convection. Alaoui et al. [35,36] did an experimental investigation using the prismatic LIB and obtained improved thermal management for the batteries. On the contrary, forced air conditioning resulted in significantly less heat dissipation than the TEC, which enabled the battery to function within an appropriate and consistent temperature range. Luo et al. [37] presented a BTMS with vapour chambers and TECs to enhance battery thermal behaviour. To forecast thermal performance under various cooling conditions, a full fluid-thermal-electric multiphysics numerical model was created for the proposed system. Results indicated that thermoelectric coolers and vapour chambers considerably reduced battery maximum and temperature differential. As air convection heat transfer coefficient and coolant flow rate rise, maximum temperature drops while temperature differential increases. The ideal air and water cooling parameters showed that when thermoelectric cooler input current rose, maximum temperature and temperature differential decreased first and subsequently increased. The recommended air convective heat transfer coefficient, coolant flow rate, and input current are 50 W/(m2·K), 0.04 m/s, and 1.5 A, resulting in a maximum temperature of 39.83 °C and a temperature differential of 5.97 °C. Using experimental data, the three-dimensional thermal model of a battery with TEC was developed and calibrated by Liu et al. [30]. Implementing TEC cooling decreased the maximal battery temperature from 31.7 °C to 26.1 °C. Negi and Mal [38] presented a technique for cooling batteries that used Thermoelectric cooling driven by PV with MPPT. The average temperature decrease of the BTMS was 5.6 °C. The study also developed a simulation model to examine a copper enclosure equipped with four Peltier devices positioned on each of the four walls, which enclose the battery pack. The average temperature drop here was 6.1 °C.

## 2 Novelty of the present review

This review article distinguishes itself in the field of BTMS by specifically concentrating on thermoelectric cooler (TEC)-based solutions for LIBs, an area that has not been thoroughly examined before. This study offers a comprehensive examination of TEC-based BTMS topologies, such as hybrid, air-cooled, PCM-cooled, liquid-cooled, and heat pipe-cooled systems. Unlike other literature that covers a wide range of BTMS technologies or particular research investigations, this paper provides an in-depth analysis. The design, performance, and practical applications of each configuration are thoroughly analysed, providing readers with a comprehensive grasp of the present status and promise of TEC technology. In addition, this study provides a comprehensive examination of heat sink designs, with a special focus on the impact of fin shape, thickness, and arrangement on TEC performance. This feature is crucial for achieving optimal thermal management; however, it is typically neglected in other reviews. In addition, the research examines advanced control techniques, including machine learning-based and model predictive control, to provide creative methods for improving the efficiency and efficacy of TEC-based BTMS.

This review addresses novel aspects and provides a forward-looking perspective on future research directions. It fills a significant gap in the literature and offers valuable insights and guidance for researchers and engineers in the field. The comprehensive and focused approach employed in this paper ensures that it serves as a critical resource, setting it apart from other published works and making a significant contribution to the advancement of TEC-based thermal management systems.

## 3 Working principle

The fundamental concepts behind the operation of the thermoelectric effect are the Seebeck effect and the Peltier effect. A phenomenon that results from a thermal gradient between two materials or semiconductors, the Seebeck effect was originally noticed in 1821. This disparity results in a variation in voltage between the two compounds.

The Peltier effect, which was initially observed in 1834, pertains to the outcome of an electric current traversing dissimilar materials. This causes an exothermic reaction to take place at the interface of the materials, as well as the generation of irreversible Joule heat. The assessment of the TEG materials is conducted using the merit figure of ZT. This figure is determined by three fundamental physical qualities, namely, the electrical conductivity, Seebeck coefficient, and thermal conductivity, which are associated with the features of N-type and P-type materials [39,40].

**Fig. 1:**
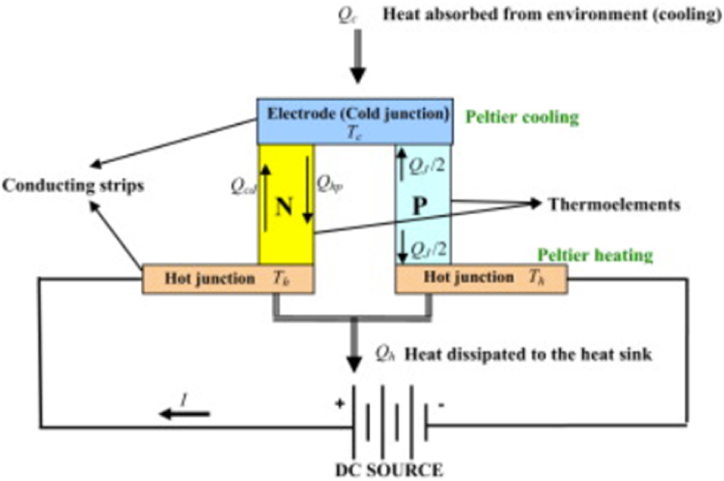
Diagram illustrating the working of a TEC [45].

**Fig. 2:**
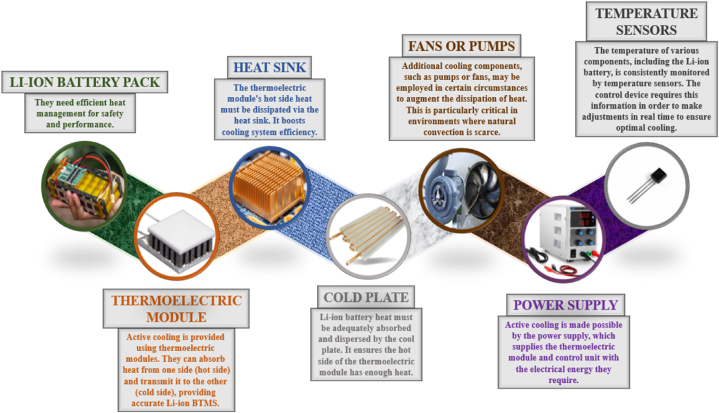
Components required for a typical thermoelectrically cooled lithium-ion BTMS setup.

A typical TEC consists of numerous P-type and N-type semiconductor junctions that are electrically coupled in series via metallic interconnects, usually made of copper. These junctions are thermally connected in parallel, creating a single-stage cooler [41]. When a DC power supply of lower voltage is connected to a TEC, thermal energy is transferred from one end of the cooler to the opposite end. Consequently, one side of the TEC gets cooled while the other side is heated. Fig. 1 illustrates a TEC module that functions as a thermoelectric refrigerator. The passage of electrical current occurs from the N-type element to the P-type element inside this module [42]. The cold junction's temperature (T_C_) lowers as transmission of heat occurs from the surroundings to the cold junction at a reduced temperature. When electrons in a transport system travel across a cold junction, they change energy levels, going from a lower one in the P-type component to a higher one in the N-type component. Concurrently, the heat that has been absorbed is transferred by the transport electrons to the heated junction, where the temperature is represented as T_H_. The Peltier effect occurs when the electrons in a P-type semiconductor return to a lower energy state, while the heat is dispersed across the heat sink. A voltage referred to as the Seebeck voltage is generated when a temperature difference exists between the cold junction and heated junction of P-type and N-type thermoelements. This voltage is directly proportional to the difference in temperature [43,44] (see Fig. 2).

The Peltier effect is a phenomenon that is taken into consideration in TEC systems, while the Seebeck effect is a phenomenon that is regarded in TEG systems. The TEC has been widely used in residential cooling and solar energy system batteries. Many research studies have extensively used the thermal energy control TEC system integrated inside the BTMS of EVs. The condition indicated above is achieved by supplying the appropriate electrical energy to both ends of the TEC to establish a temperature gradient between its cold and hot surfaces. In contrast, the valuation of the electrical energy contribution and the produced materials within the context of TEC is contingent upon the magnitude of the temperature differential. The continuous provision of energy to the TEC is unattainable owing to the escalating impact of joules. The Joule heating effect refers to the heat created by the passage of electric current in a metallic conductor. This phenomenon entails the application of heat to the cathode filament of an X-ray tube, increasing the temperature of its previously cool surface. Hence, attaining optimal operational efficiency for TEC necessitates using a suitable control mechanism [44]. Integrating TEC and TEG into a single model has emerged as a promising technological advancement in refrigeration applications and BTMS. TEGs, which operate based on the Seebeck effect, have many advantages. One such benefit is the ability of TEGs to collect waste heat from the hot surface of TECs and convert it into electrical energy [46]. This generated energy is feedback energy for TECs, optimizing their performance. Additionally, TEGs contribute to the overall lowering of temperature in the battery pack of EVs, enhancing their efficiency and functionality [[47], [48], [49]].

## 4 Experimental studies

A thermoelectric cooler is a solid-state semiconductor device that offers a multitude of advantages. It operates noiselessly, has minimal impact on the environment, and requires no additional costs for operation or maintenance. A TEC unit comprises P-type and N-type thermoelectric (TE) legs that are electrically linked in series and thermally connected in parallel. When compared to a standard refrigerator, TEC consumes less voltage and current. Consequently, the performance is contingent upon the TEC power supply. Furthermore, because of its tiny size in comparison to usual refrigeration and power requirements, the traditional refrigeration system requires far less power density than TEC. Nevertheless, this diminished thermal efficiency may be advantageous in several small-scale applications, particularly in cases where there is a surplus of heat energy or a requirement to regulate excess heat without compromising the primary system's performance. Therefore, TEC might serve as a feasible choice for the battery temperature control system in electric/hybrid vehicles.

A standard configuration for a thermoelectrically cooled lithium-ion BTMS would consist of a LIB pack, a thermoelectric module, a heat sink, a cold plate, fans (or pumps), a power supply, and temperature sensors. The function of each component is illustrated in Fig. 1.

**Fig. 3:**
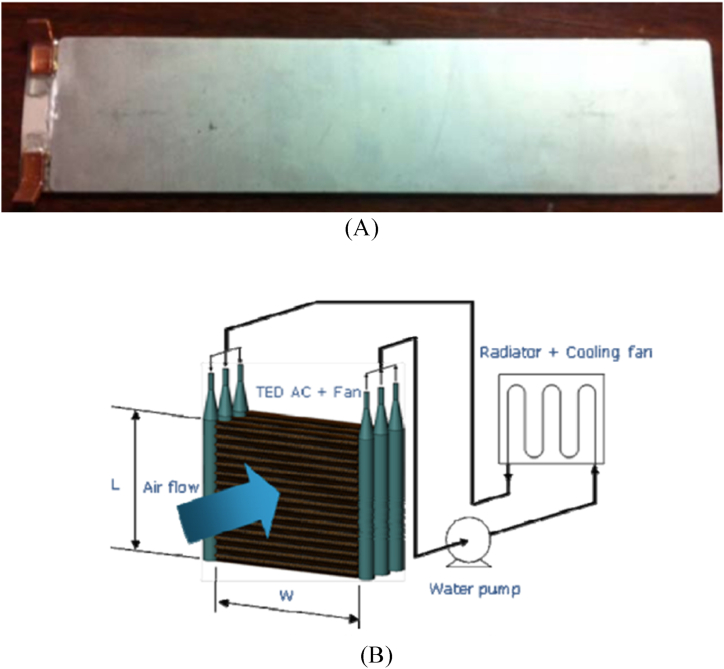
(A) TED construction for a battery cooling module [50] (B) Thermoelectric Modules (TEMs) integrated into an air-cooled system [50].

The thermoelectric battery cooling system developed by Kim et al. [50] included a thermoelectric cooling module (TEM) (see Fig. 3 (A)), a pump, a radiator, and a cooling fan as illustrated in Fig. 3 (B). A thermal design analysis was performed in this study on a 1 kW thermoelectric battery cooler in order to optimise the coefficient of performance (COP) and devise an appropriate method for regulating the temperature of the battery. Following that, numerous prototypes of a 1 kW thermoelectric battery cooler were produced and tested, having been designed and developed via thermal design analysis. The prototype thermoelectric modules used dimple fins as well as louver fins on the air supply end. A water-cooling jacket was employed to efficiently cool the hot side of the thermoelectric battery cooler. Experimental trials were conducted on the prototype 1 kW thermoelectric battery coolers inside a controlled environment of consistent temperature and humidity. The battery cooler's COP ranged from 0.44 to 0.70 for chilling and from 0.92 to 1.28 for heating. The study revealed that the efficiency of the airside heat transfer fins had a significant impact on the 1 kW thermoelectric cooler's overall COP.

## 5 Governing equations

### 5.1 Li-ion battery profile

The thermal energy produced by the battery encompasses the heat created via electrochemical reactions, joule heating, polarisation heating, and side reaction heating [51]. This may be quantified using Eq [1].

[1] $$Q=Q_{r}+Q_{j}+Q_{p}+Q_{s}$$

Q represents the overall amount of heat that the battery produced. $Q_{r}$ denotes the heat produced by the electrochemical reaction during the charging and discharging of a battery, particularly during the process of Li ion insertion and removal between the anode and cathode materials. $Q_{j}$ represents the heat generated due to the internal resistance of the battery. $Q_{p}$ represents the heat generated by the internal resistance caused by polarisation during the charging and discharging process. The heat generated throughout the electrolyte's decomposition and reaction, the anode and cathode materials' thermal decomposition, and the separator's decomposition is denoted by $Q_{s}$.

Directly measuring the heat produced by a LIB in the experiment was challenging due to the impact of the surroundings and transmission heat loss. The computation approach described by Bernardi et al. [51] was used in this study. A theoretical formula was presented for the rate of heat production [52,53] under the premise that heat generation within the LIB is uniform. The formula is simplified as shown in Eq [2].

[2] $$Q=I\left(E-U\right)-\text{IT}\frac{\partial E}{\partial T}$$

The variables in the equation are as follows: I represents the current while the Li-ion battery is being charged or discharged, E represents the open circuit voltage, U represents the closed-circuit voltage, and T represents the temperature of the battery's surface, measured in kelvins. The right-hand component of the equation indicates the heat generated by irreversible reactions, such as joule heat and electrochemically polarised heat. This heat may be quantified as $I^{2}R_{\mathrm{int}}$, where $R_{\mathrm{int}}$ denotes the total internal resistance of the LIB. The second component corresponds to the thermal energy generated via reversible process, and in the case of a LIB, it typically assumes a reference value of 0.042 [52]. Once the values are entered into the formula, the result (Eq. [3]) is obtained.

[3] $$Q=I^{2}R_{\mathrm{int}}-0.042I$$

Eq [4]. provides an approximation of the total heat generated in the mode of a battery pack comprising n cells connected in series [54]. The same methodology was employed, with the assumption that the heat coefficient remained constant on the surfaces of the battery cells on all sides. and an equivalent moderate forced convection value with air.

[4] $$Q_{\text{pack}}=\text{nQ}$$

The energy needed to charge the battery from an initial state of charge (SOC_1_) to a final state of charge (SOC_2_) is denoted as $E_{\text{charge}}$ and may be calculated using Eq [5].

[5] $$E_{\text{charge}}=E_{\text{battery}}+E_{\text{charge},\text{rev}}+E_{\text{charge},\text{irrev}}$$

$E_{\text{charge},\text{rev}}$ represents the energy associated with the entropy change; $E_{\text{battery}}$ denotes the energy contained in the battery; and $E_{\text{charge},\text{irrev}}$ signifies the energy lost due to polarisation. Similarly, the total energy used to discharge the battery from SOC_2_ to SOC_1_ is represented by Eq [6].

[6] $$E_{\text{battery}}=E_{\text{discharge}}+E_{\text{discharge},\text{rev}}+E_{\text{discharge},\text{irrev}}$$

where $E_{\text{discharge}}$ represents the electrical activity executed by the battery, $E_{\text{discharge},\text{irrev}}$ denotes the energy dissipated due to polarisation, and $E_{\text{discharge},\text{rev}}$ corresponds to the entropy change. In final, Equation [7] represents the total energy loss throughout the cycle.

$$E_{\text{losses}}={E_{\text{charge}}-E}_{\text{discharge}}$$

[7] $$=\left(E_{\text{charge},\text{rev}}+E_{\text{discharge},\text{rev}}\right)+\left(E_{\text{charge},\text{irrev}}+E_{\text{discharge},\text{irrev}}\right)$$

The Eq [8]. represents the overall heat loss [55].

[8] $$Q_{\text{battery}}=\frac{E_{\text{losses}}}{\Delta t}$$

where Δt represents the time interval necessary to go from SOC_1_ to SOC_2_. When the current is strong, the main factor contributing to heat generation is irreversible heat, which includes ohmic resistance heat, polarisation resistance heat, and tab resistance. This phenomenon has been seen in previous studies [[56], [57], [58]]. The magnitude of heat dissipation is significantly impacted by the lumped internal resistance value. This refers to the measurement of the resistivity of the individual components of the battery. $R_{\mathrm{int}}$ is also impacted by physical interactions (such as the connection between the current collector and the active material), the structural characteristics of the electrode (such as its thickness and size), and internal design factors.

### 5.2 Peltier thermoelectric module

A Peltier TEM has many semiconductor thermocouples, sometimes referred to as thermoelectric legs, that are positioned between two ceramic substrates. The ceramic substrates provide exceptional thermal conductivity to ensure low temperature dissipation across the substrate layer. The process of actively removing heat is accomplished by applying direct current (DC) via a set of thermocouple 'legs' which are thermally parallel and electrically coupled in series. A thermocouple leg consists of a semiconductor material that is either n-type or p-type. Excess electrons flow from p-type to n-type materials, where they leap to a higher state of energy and absorb thermal energy. As they progress through the lattice of the material, electrons transition from the n-type to the p-type, thereby reducing their energy level and emitting heat energy towards the heat sink [59,60]. The heat that a TEM absorbs ($Q_{C}$) and dissipates ($Q_{H}$) may be described using Eq. [9,10], where ΔT represents the temperature differential between the device's hot and cold sides ($\Delta T={\;T}_{H}-T_{C}$). The electrical resistance and the Seebeck thermal conductance are denoted by R, α, and K, respectively. The device's thermoelectric leg count, n, is the number of pairs. As shown in Eq. [11], the electrical power, $P_{\text{electrical}}$ required to run the TEM denotes the difference between the heat absorbed (QC) and the heat dissipated ($Q_{H}$).

[9] $$Q_{C}=2n\left(\alpha T_{C}-\frac{1}{2}RI^{2}-K∙\Delta T\right)$$

[10] $$Q_{H}=2n\left(\alpha T_{C}+\frac{1}{2}RI^{2}-K∙\Delta T\right)$$

[11] $$P_{\text{electrical}}=Q_{H}-Q_{C}=2n\;\left(\alpha I\Delta T+RI^{2}\right)$$

The fabrication of TEM entails the placement of n semiconductor legs amongst two ceramic substrates. The thermal resistance of N thermoelectric modules (TEMs), denoted as $\theta_{\text{TEM}}$, is the sum of the thermal resistance of the ceramic substrate ($\theta_{\text{cer}}$) and the thermal resistance of the semiconductor legs ($\theta_{\text{semi}}$), when no power is supplied. The thermal resistance of the connecting conductors is disregarded. The thermal resistances are denoted by Eq. [12,13], and [14].

[12] $$\theta_{\text{cer}}=\frac{t_{\text{cer}}}{nK_{\text{cer}}∙A_{\text{cer}}}$$

[13] $$\theta_{\text{semi}}=\frac{t_{\text{semi}}}{K_{\text{semi}}\left(2∙n∙N∙A_{\text{semi}}\right)}$$

[14] $$\theta_{\text{TEM}}=2\theta_{\text{cer}}+\theta_{\text{semi}}$$

The variables $K_{\text{semi}}$, $t_{\text{cer}}$ and $A_{\text{cer}}$, represent the thermal conductivity, thickness, and cross-sectional area of the ceramic substrate and the semiconductor leg, respectively.

## 6 TEC based hybrid BTMS

Various heat dissipation strategies in BTMS have been suggested by domestic and international experts. Xiaoyu Na et al. [61,62] developed a simplified calculation model for reverse-ventilated battery pack cooling and shown that this technique efficiently reduces the maximum interior battery pack temperature while also reducing the local range of temperatures. However, air cooling cannot effectively manage the temperature in hot weather. Liquid cooling employs liquid to cool the power battery, classified as active or passive [63]. Chunrong Zhao et al. [64,65] created a serpentine pipe within a cylindrical battery module. Under 5C discharge, the numerical simulation demonstrates that 2.2 °C lowers the battery's maximum temperature range. Bittagopal Mondal et al. [66,67] created a vortex-generating device within a flat tube. The investigation revealed that the inclusion of the eddy current channel significantly enhanced heat transmission in the cooling channel, resulting in a notable 10 % decrease in the maximum battery pack temperature. The two liquid cooling systems have greater cooling channel design and material selection requirements and need additional optimization. The EV BTMS, initially introduced and patented by Al-Hallaj et al. [68], incorporates PCM. By absorbing heat during PCM melting, battery cells or modules can be submerged directly in PCM, thereby regulating the battery's temperature [[69], [70], [71]]. Nevertheless, due to the limited thermal conductivity of paraffin wax (PW), the areas of contact between the battery and the PCM may experience excessively high temperatures before other components dissolve, resulting in a degradation of system performance [72,73]. Semiconductor-based TEC cooling has thus gained significant interest due to its quick activation time, simple design, and capability to cool rapidly with sudden high currents. Typically, it is integrated with one or more other cooling techniques [74]. Luo et al. [75] achieved the ideal operating temperature of lithium-ion batteries by integrating thermoelectric cooling with water and air cooling systems. A hydraulic-thermal-electric multiphysics model was developed to evaluate the system's thermal performance. The impact of several cooling factors, including the input current of the thermoelectric cooler (TEC), the air convection heat transfer coefficient, and the cooling water flow rate on thermal performance is thoroughly examined by numerical simulations. The findings indicated that incorporating thermoelectric cooling into battery thermal management enhances the cooling efficacy of conventional air and water cooling systems. Furthermore, the cooling power and coefficient of performance (COP) of thermoelectric coolers initially rise and subsequently decline with increasing input current. With an air convection heat transfer coefficient of 50 W m−2 K−1, a water flow rate of 0.11 m/s, and a TEC input current of 5 A, the battery thermal management system achieves optimal thermal performance, yielding a maximum temperature of 302.27 K and a temperature differential of 3.63 K. Hao et al. [76] conducted a dimensional analysis using the thermoelectric conversion model to optimise the thermoelectric capacity. The study also investigated the Thomson effect of thermoelectric devices, which was shown to be insignificant for minor temperature differences [77]. In 2010, Bartek et al. created a thermal management system for a power battery pack using TED technology. They then installed this system on SAM EV-II, a vehicle produced in large quantities [78]. The following section will look into the various TEC-hybrid models for Li-ion battery cooling systems based on various simulations and experiment-based research performed in the field.

## 7 Air cooled thermoelectric BTMS

Air cooling, a thoroughly studied and often used method, has been thoroughly examined and is frequently utilized in commercial applications [22,79]. Air cooling is considered one of the most efficient methods for achieving cooling efficiency in BTMS due to its comprehensive cost, tightness, and other aspects [[80], [81], [82]]. An air-cooled BTMS is a direct and efficient approach to managing heat generated inside battery packs, particularly in EVs with limited design space [83]. Some research indicates that forced air conditioning struggles to achieve the desired cooling effect when mass battery packs are discharged at high velocities [84]. Innovative BTMS designs are highly sought in both the academic and industrial sectors. The combination of air cooling and thermoelectric cooling is a highly desirable method for battery cooling in the scientific community. Hameed et al. [85] presented a hybrid BTMS integrating thermoelectric materials with pushed air. The research created A single unit by combining the TEC and TEG. TECs have traditionally been used in BTMS. However, the recent inclusion of TEGs enables the conversion of wasted heat from the TEC's hot surface into a reverse voltage that supplies power to both the TEC and TEG. The implemented BTMS was used in the present investigation to reduce an individual battery cell's maximum battery surface temperature (Tmax) by roughly 7 °C. A TEG in conjunction with forced convection (F-C) was introduced by Li et al. [39] as a viable and efficient cooling mechanism for a BTMS. The TEG cooling system is superior to natural convection cooling, F-C cooling, and comparison-based cooling. With regard to temperature reduction, this system exhibits a capacity of 16.44 % at a 3C discharge rate. Significant temperature regulation is possible with the coupled F-C and TEG cooling system despite the relatively high discharge rate. Lyu et al. [86] created a BTMS that included forced air cooling, thermoelectric cooling, and liquid cooling. By means of forced air cooling, heat was withdrawn from the condenser end of the thermoelectric liquid case. Elaborate tests are conducted on a simulated EV battery system. The experimental findings demonstrated a favourable cooling impact, accompanied by a moderate degree of power dissipation. In addition, the experimental trial revealed that the surface temperature of the battery decreased by approximately 43 °C (from 55 °C to 12 °C) when a single cell with a copper holder was subjected to a TEC-based water-cooling system, with a heater provided with 40 V and the TEC module supplied with 12 V. Esfahanian et al. [87] implemented an air flow system consisting of fans, fins, heat sinks, and thermoelectrics to enhance temperature uniformity and decrease the maximum cell temperature in the BTMS of a hybrid electric bus. For this investigation, a battery pack comprised of 12 smaller packs, each containing 14 porch cells arranged in series, was utilized. The battery was customised specifically for the Vehicle, Fuel, and Environments Research Institute (VFERI)-manufactured hybrid electric bus. The numerical findings demonstrated that the battery temperature should remain below 35 °C and the temperature differential (Δ T) between cells should be kept within 5 °C during periods of rapid charge/discharge rates and when the ambient temperatures are above 40 °C. The investigations that have been conducted on air-cooled thermoelectric BTMS are summarised in Table 1.

**Table 1:** An overview of the thermal management of batteries integrating a variety of air-cooled TEC-Based techniques.

Work	Layout	Battery type	Ambient temperature	Heat load, C-rate	Air speed	Key findings
Hameed et al. [85] (2023)		LiFePO4 cell	25 ± 1 °C	Discharge rate = 1C	0.6594 m/s	• T_max_ decreases from 38 °C to 33.1584 °C with free air convection. • T_max_ experiences a reduction from 38 °C to 32.7836 °C as a result of forced air convection.
Li et al. [39] (2019)		18650-type batteries	28.82 °C	Discharge rate = 1C, 1.5C, 2C, 2.5C and 3C.	–	• At 3 C discharge rate, the T_max_ is 65.43 °C, much lower than the F-C (70.52 °C) and N-C (78.30 °C) cooling models. • The FC + TEG-40 °C cooling type was the best BTMS for energy usage and temperature control.
Lyu et al. [86] (2019)		18650 Li-ion battery	20–25 °C	CSH-02120 heaters with rated power at 20 W	–	• TEC-based water-cooling system for a single cell case with copper holder decreases surface temperature of the battery by 43 °C (from 55 °C to 12 °C) using 40 V to the heater and 12 V to the TEC module.
Esfahanian et al. [87] (2023)		Battery pack created for hybrid electric bus presented by VFERI	40 °C	Discharge rate = 4C	–	• Air cooling BTMS using TEC can achieve the top safety limit (35 °C) and lower the cell's worst-case Δ T to 6 °C. • With a twin pair of blowers and two heat sinks, cell heat may be removed and the thermoelectric's hot surface can be kept below 53 °C.

## 8 PCM cooled thermoelectric BTMS

Nowadays, PCM is widely used in BTMS because of its superior temperature uniformity and heat absorption [88,89]. However, the two biggest disadvantages of PCM were its poor thermal conductivity and limited thermal storage capacity [90]. While some researchers have looked at using metal mesh, porous media, and nanomaterial additions with high thermal conductivity to improve the performance of PCM, difficulties persist regarding the costly and intricate production of high-performance materials [91]. In order to recover latent heat in PCM, additional active thermal management techniques are frequently implemented, resulting in a hybrid BTMS [92]. Liu et al. [93] proposed a PCM and TEC based BTMS to counteract the rapid fluctuations in temperature and poor temperature uniformity during high battery discharge rate. Zhang et al. [94] examined the increase in temperature and the uniformity of the 100Ah TAFEL-LAE895 type ternary lithium-ion power battery via charging and discharging trials at various rates. Paraffin was used to decrease the battery's surface temperature. Simultaneously, extended graphite (EG) was incorporated into the composite phase change material (CPCM) to improve its thermal conductivity and viscosity, and to reduce its fluidity upon melting. The thermal management impact is directly affected by the PCM's reduction in heat storage capacity as the graphite concentration increases. Therefore, for efficient heat dissipation, this research incorporated heat pipe and semiconductor refrigeration technology to convey heat from the interior CPCM to the thermoelectric cooling sheet. The findings indicate that the temperature on the battery surface may be effectively controlled within an acceptable range during high-rate discharge. The battery's temperature consistency is enhanced, resulting in energy conservation.

Liao et al. [95] introduced a hybrid active-passive full-temperature BTMS that integrated PCM and TEE to regulate the temperature of LIBs operating in harsh environments in the Central and Southern China region (313.15 K when temperatures are high and 268.15 K when temperatures are low). The findings indicated that when CPCM and TEE were utilized in conjunction, it was possible to regulate the maximum battery temperature to an extent below 318.15 K in a hot environment at a high discharge rate of 3C. Additionally, the maximum difference in temperature during the battery module's discharging process could be maintained within 3 K. Concerning the preheating process, the maximum temperature difference could be regulated within 5 K, and the RTR (Rate of Temperature Rise) could range from 0.808 to 1.33 K/min. When subjected to ambient conditions ranging from 268.15 to 313.15 K, the batteries could be maintained in a comfortable thermal environment using the full-temperature BTMS between 293.15 and 318.15 K. A hybrid BTMS was presented by Liu et al. [96], which integrates TEC and PCM. A comparative analysis revealed that incorporating PCM resulted in improved temperature uniformity, a temperature difference of less than 6 °C was maintained, and CPCM with a higher thermal conductivity was deemed highly appropriate for the hybrid BTMS. Specifically, at 1200s, the model incorporating 30 % EG/RT44HC exhibited a maximum temperature 10.3 °C lower than that constructed using pure RT44HC. An increase in fill thickness for CPCM resulted in an extension of the temperature control time; however, it also led to a decline in the efficacy of TEC. In this particular model, the orientation of the fins should be positioned normal to the battery's direction. This was done to facilitate the efficient and quicker transmission of heat from the batteries to the fins, which possessed a higher thermal conductivity. Song et al. [97] used semiconductor thermoelectric devices as well as PCMs to regulate the temperature of 48-V 80 Ah LIB packs for base stations. Findings show that a semiconductor thermoelectric device and PCMs are capable of maintaining the optimal temperature range for outdoor base station standby battery packs for 4.4 days at 323 K after cooling and 3.52 days at 263 K after heating. As power rose, cooling/heating time and heat preservation reduced. Based on the semiconductor thermoelectric devices' stability and hot-end heat dispersion, 200 W was the optimal cooling power. To reduce heating frequency and usage, a 200-W battery pack was recommended. At ambient temperatures between 333 K and 253 K, cooling/heating time reduced, and heat preservation duration rose as PCMs' phase transition temperature approached. Luo et al. [98] introduced a BTMS that is integrated with TECs and PCMs in the fin framework to regulate the temperature of the battery. The thermal performance of the BTMS was investigated in two scenarios using a transient thermal-electric-fluid multi-physics field numerical model. The maximal temperature and PCM liquid percentage were decreased as a result of the increased TEC input current, fin length, and thickness. The temperature differential was diminished by fin length; however, fin thickness and TEC input current had negligible effects. The ideal fin length and thickness were 7 mm and 3 mm, respectively, as indicated by numerical results. The maximum temperature, temperature differential, and PCM liquid percentage of Case 1 were 315.10 K, 2.39 K, and 0.002, respectively, when the TEC input current was 3 A. In contrast, Case 2's values are 318.24 K, 3.60 K, and 0.181. Due to the fact that Case 1 had a larger battery pack-to-TEC distance and fewer TECs, it performed better than Case 2. As the battery discharge rate increases, the TEC input current should be increased to maintain the battery temperature. The studies completed on PCM-cooled thermoelectric battery thermal management systems are presented in Table 2.

**Table 2:** Overview of a variety of PCM-Cooled TEC-Based techniques and their integration into battery thermal management.

Work	Layout	Battery type	Ambient temperature	Heat load, C-rate	Type of PCM	Key findings
Liao et al. [95] (2021)		16 21,700 power LIBs	40 °C and −5 °C	Discharge rate = 3C	RT44HC and CPCM	• T_max_ of RT44HC may be decreased by 15 K at 2 mm and 7 K at 4 mm when TEE's operating current is 1.2A. • CPCM reduces T_max_ by 3 K and Δ T by 2 K compared to RT44HC.
Liu et al. [96] (2022)		LiFePO4 type battery	30 °C	Discharge rate = 3C	RT44HC and CPCM	• As PCM improves temperature uniformity, the Δ T was less than 6 °C. • T_max_ of the CPCM model with 30 % EG/RT44HC was 10.3 °C below pure RT44HC.
Song et al. [97] (2018)		48 V and 80Ah battery pack	40 °C	Discharge rate = 1C	n-octadecane	• The highest Δ T of the pack during cooling or heating was below 5 K. • The optimal cooling and heating power was 200 W. • T_max_ was maintained below 312 K.
Liu et al. [93] (2023)		–	30 °C	Discharge rate = 4C	–	• When current is 2A, the battery functions best below 40 °C. • Constant cooling with a higher TEC current degrades battery temperature uniformity, PCM utilization, and TEC cooling efficiency. • The discharge phase temperature gradient is always smaller than 5 °C with delayed cooling with 2A TEC current at 80 % PCM melting rate. • The discharge at 4C results in exceptional battery temperature uniformity and a temperature differential of 2.5 K.
Jiang et al. [99] (2019)		18650 battery 3 × 5 array	15 °C	Heating power of battery module = 45 W (3 W for each battery)	Copper foam-PCM composite	• With a 6 W battery heating power, TEC took 5335s to achieve 50 C, compared to 930s for natural convection and 1275s for liquid cooling.
Alghamdi et al. [100] (2023)		LIB model 26500	25 °C	Heaters with rated power at 2–15 W	Paraffin	• On adding the thermoelectric module, the battery's average temperature (T_ave_)dropped from 85 °C to 76 °C. • T_ave_ dropped to 65 °C after installing circular aluminum fins. • T_ave_ obtained with the axial fin design was 48 °C.

## 9 Liquid cooled thermoelectric BTMS

Pesaran et al. [101,102] recognized the need for thermal management of EV and HEV batteries in the early 2000s. Ensuring an even distribution of temperature and providing an ideal operating environment for the battery modules were both critical aspects of this process. Based on the research, it is essential to use liquid based BTMS for EVs and series HEVs. Since 2010, the rapid advancement of LIB and EV technology has led to EV manufacturers' widespread use of liquid-cooling BTMS [2]. This is primarily due to the excellent thermal properties of water-based coolants, which provide significant promise for managing heat in EVs [103]. When compared to air, water has a greater specific heat and thermal conductivity. Water and oil are utilized as coolants in many systems for cooling. As a result, water plays an essential function in a variety of cooling systems, including the machining system, electronic components, and engine cooling system [[104], [105], [106]]. Water is employed in many processes in the battery system. In order to prevent an electrical short circuit, the water is isolated from the primary heat source, which includes the cooling surfaces, tubes, and jacket [107,108]. Water and oil are primarily utilized in cooling systems. In addition, there has been significant interest in using ferrofluid as a coolant in thermal devices to enhance heat transmission. Ferrofluids possess characteristics such as viscosity and conductivity, and they exhibit sensitivity to magnetic fields. Ferrofluid can improve heat transfer.

Lyu et al. [31] introduced a novel battery pack configuration comprising battery cells, copper battery carriers, an acrylic battery container, and a liquid cooling medium. This battery unit was integrated with a BTMS that utilized liquid and air circulations in addition to TEC. Initial optimization of the fundamental design was performed on a single cell. The efficacy of the system was subsequently verified through the battery pack experiments. In comparison to liquid cooling alone, the proposed system achieved an approximate 20 °C lower temperature during the 40 V test. During the 5000 s, 30V power supply test, the temperature of the battery charge remained below 30 °C. Furthermore, when the battery pack was being continuously discharged with a 50 V input, its temperature decreased to below 60 °C for a duration of 3000 s. This temperature is regarded as an extreme circumstance for battery functioning. Sirikasemsuk et al. [109] investigated the thermal properties of an 18650 type LIB pack with a thermoelectric module that used ferrofluid as a coolant. The effects of significant factors such as hot and cold side flow rates (0.030.05 m^3^/h), provided voltage via thermoelectric (812 V), coolant types (ferrofluid and deionized water), and ferrofluid concentrations (0.005 %0.015 % by volume) on the cooling performance of the battery pack were tested. It was discovered that the TEC system has a substantial impact on the pack's cooling performance and keeps the battery temperature lower than 30 °C. Increasing the flow rates on both the cold and hot sides of the battery will potentially lower the average battery cell temperature by 3 °C–5 °C. This will result in a temperature uniformity of less than 3 °C. Furthermore, as compared to deionized water, ferrofluid concentration dramatically lowered average battery cell temperature. Table 3 summarises the investigations that have been conducted on liquid-cooled thermoelectric BTMS.

**Table 3:** Overview of a variety of liquid-cooled TEC-Based techniques and their integration into battery thermal management.

Work	Layout	Battery type	Ambient temperature	Heat load, C-rate	Flow speed	Key findings
Lyu et al. [31] (2021)		21700 LIB	20–25 °C	CSH-102100 heater with rated power at 100W	4 L/min	• Compared to using solely liquid cooling, the suggested approach achieved around 20 °C lower in the 40 V test.
Sirikasemsuk et al. [109] (2020)		18650-type LIB pack	25 ± 1 °C	Current rate = 1A, 2A and 4A	0.03–0.05 m^3^/h	• Battery cell temperatures remained below 40 °C due to liquid cooling circulation. • Increased cold and hot side flow rate lowered battery cell temperature by 3–5 °C, resulting in a uniform temperature below 3 °C in the cooling pack.

## 10 Heat pipe cooled thermoelectric BTMS

Both a heat pipe (HP) and a TEC have been the subject of investigation [110,111]. The HP has a significant thermal conductivity in the longitudinal direction and demonstrates isothermal characteristics in the heat-conductive direction, while the TEC is distinguished by its refrigeration capability, rapid heat transfer, small size, and high reliability. When Joshua et al. [112] implemented the HP in BTMS, they discovered that it could regulate the Li-ion battery's temperature more effectively than the conventional liquid cooling system. Deng et al. [113] constructed a BTMS by combining an L-shaped HP with an aluminium plate and demonstrated that as the ambient temperature rises, both the heat dissipation rate from the HP and the increase rate in battery temperature reduction. However, the HP is incapable of adequately dissipating battery heat when the discharge rate is excessive. While rapid heat dissipation of the battery is possible through the TEC cold side, dissipating the generated heat on the heated side under natural conditions is more challenging. Consequently, a BTMS that integrates heat pipe and TEC cooling may be of interest in the current situation.

Zhang et al. [51] conducted an investigation whereby they devised a BTMS that integrated TEC and HP technologies. The researchers evaluated the system's performance and examined the battery's patterns of surface temperature rise under varying discharge rates. The findings indicate that the heat pipe adequately regulated the battery temperature within the designated range, as shown by a moderate discharge rate. In order to achieve a high discharge rate, the incorporation of an additional thermoelectric cooler was deemed necessary to facilitate future enhancement. Additionally, the findings of the study indicate that the integrated system has the potential to efficiently decrease the battery's surface temperature throughout the whole range of discharge rates anticipated in the specific battery under investigation. Research on thermoelectric BTMSs cooled by heat pipes is summarised in Table 4.

**Table 4:** Synopsis of a heat-pipe-cooled TEC-Based method for battery thermal control.

Work	Layout	Battery type	Ambient temperature	Heat load, C-rate	Thermoelements	Key findings
Zhang et al. [51] (2020)		TAFEL-LAE895 100Ah ternary LIB	24.85 °C	Discharging rate = 0.5C, 1.5C, 2C, 2.5C and 3C	N- and P-type thermo-elements	• The TEC started operating at 318 K at 2C discharge rate and 303 K at 2.5C.

## 11 Comparative evaluation and real-world performance of TEC-based hybrid battery thermal management systems

In metropolitan settings, marked by frequent stop-and-go traffic, hybrid BTMS use PCM to efficiently regulate the intermittent heat produced from braking and accelerating. Phase Change Materials (PCMs) absorb and retain surplus thermal energy, so averting battery overheating and ensuring a consistent temperature distribution. This continuous temperature control safeguards the battery from thermal stress and enhances its operating lifetime [114].

Passive PCM components and liquid cooling are implemented by hybrid BTMS during rapid charging or high-speed highway transportation. This dual approach guarantees effective temperature regulation, even in the presence of severe conditions. For instance, hybrid systems that integrate Conductive Block Additives (CBA) into RT35HC PCM have exhibited substantial enhancements in thermal management. The optimised PCM, which contained a 20 % CBA mass fraction, demonstrated a thermal conductivity of 8 W/(m·K) and a heat capacity of 85 kJ/(kg·K), thereby improving performance in simulated driving and fast-charging scenarios [115].

Among the numerous design innovations of the PCM/CBA BTMS are an optimised PCM thickness of approximately 1.5 mm and a honeycomb structure that improves thermal performance and space utilization. Furthermore, the vertical water flow design enhances cooling efficacy by reducing temperature gradients. These developments accentuate the system's efficacy in real-world applications and contribute to its superior performance in comparison to other BTMS configurations.

Although innovative designs that integrate air cooling with TECs have enhanced performance, air cooling alone may be unable to sustain optimal temperatures during high-power operations [83,84]. Conversely, PCMs provide superior temperature uniformity by absorbing and releasing heat during phase transitions [88,89]. Despite the fact that PCMs improve energy conservation and temperature stability, their effectiveness may be limited by their poor thermal conductivity and limited thermal storage capacity, particularly in high-rate discharge scenarios [90,91]. Hybrid systems that incorporate PCMs with other cooling technologies have demonstrated the potential to overcome these constraints [92,93].

Due to their high thermal conductivity and specific heat, liquid cooling systems are particularly effective for large battery packs and high discharge rates [101,102]. These systems utilise fluids such as water or oil to effectively manage heat. These systems are intricate and pose challenges associated with the necessity of electrical isolation and potential leakage, despite their efficacy [103,104]. On the other hand, heat pipe cooled systems offer effective heat transfer and high thermal conductivity; however, they may not be sufficient in the presence of extreme discharge rates [110,111]. Integrating TECs with heat pipelines can improve performance, particularly at moderate discharge rates [112,113]. In general, the selection of the most suitable cooling system is contingent upon the balance of efficiency, complexity, and application-specific requirements, despite the fact that each cooling method has its own advantages and disadvantages [51].

The subsequent table (Table 5) provides a comprehensive overview of the main characteristics and performance aspects of thermoelectric BTMS that are air-cooled, PCM-cooled, liquid-cooled, and heat pipe-cooled. This information is intended to assist in the selection of the most appropriate system for various battery applications.

**Table 5:** Overview of a variety of liquid-cooled TEC-Based techniques and their integration into battery thermal management.

BTMS Type	Key Features	Temperature Range	Cooling Efficiency	Advantages	Limitations	References
Air-Cooled	Air convection + Thermoelectric Coolers (TECs)	20–28.82 °C	Tmax reduction from 38 °C to ∼32.78 °C	Simple setup; cost-effective; effective in moderate conditions	Limited effectiveness at high discharge rates; less efficient for high heat loads	Hameed et al. [85], Li et al. [39], Lyu et al. [85], Esfahanian et al. [87]
PCM-Cooled	Phase Change Materials (PCMs) + TECs	15–40 °C	Tmax reduction by up to 15 K; uniformity improvement	Enhances temperature uniformity; good for energy conservation	Limited thermal conductivity; slower response to rapid temperature changes	Liao et al. [95], Liu et al. [96], Song et al. [97], Jiang et al. [99], Alghamdi et al. [100]
Liquid-Cooled	Liquid coolant (water/oil) + TECs	20–25 °C	Temperature maintained below 30 °C; uniform temperature below 3 °C	High cooling capacity; effective at high heat loads	Potential risk of leaks; requires isolation from electrical components	Lyu et al. [31], Sirikasemsuk et al. [109]
Heat Pipe-Cooled	Heat Pipes (HP) + TECs	24.85 °C	Effective at moderate discharge rates	High thermal conductivity; effective for moderate cooling	Requires TECs for high discharge rates; limited performance at high heat loads	Zhang et al. [51], Deng et al. [113], Joshua et al. [112]

## 12 Effect of heat sink fin shape, thickness, and arrangement In TEC

**Fig. 4:**
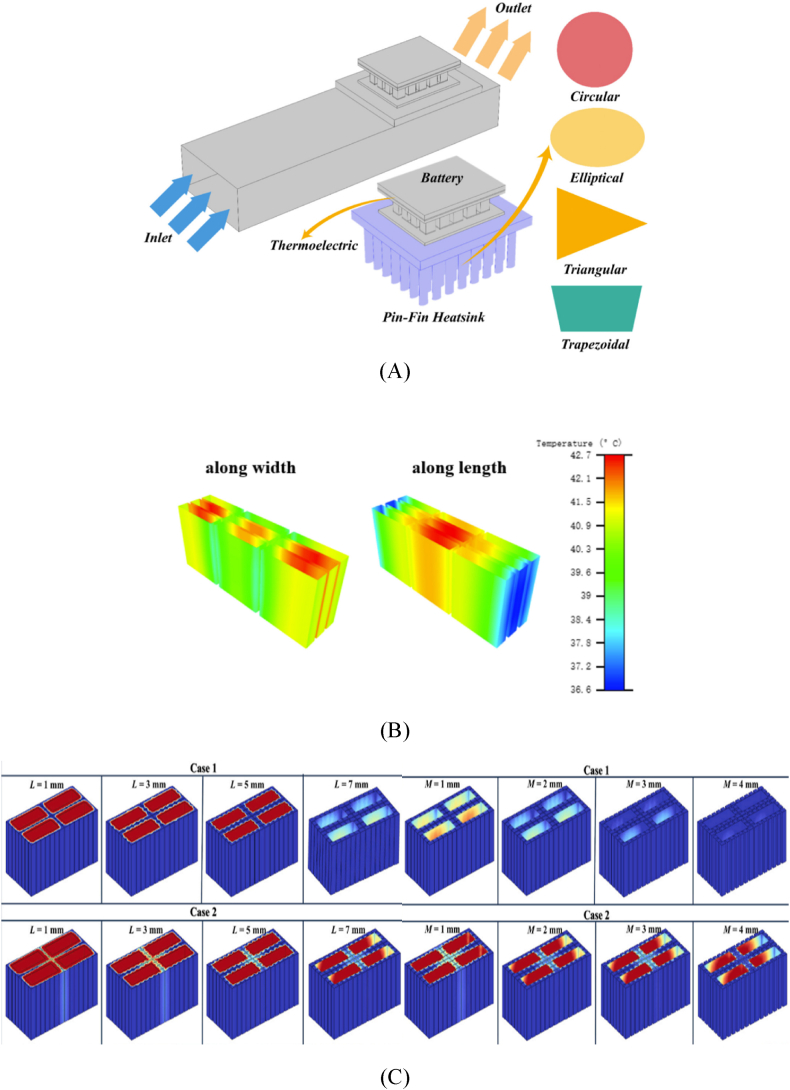

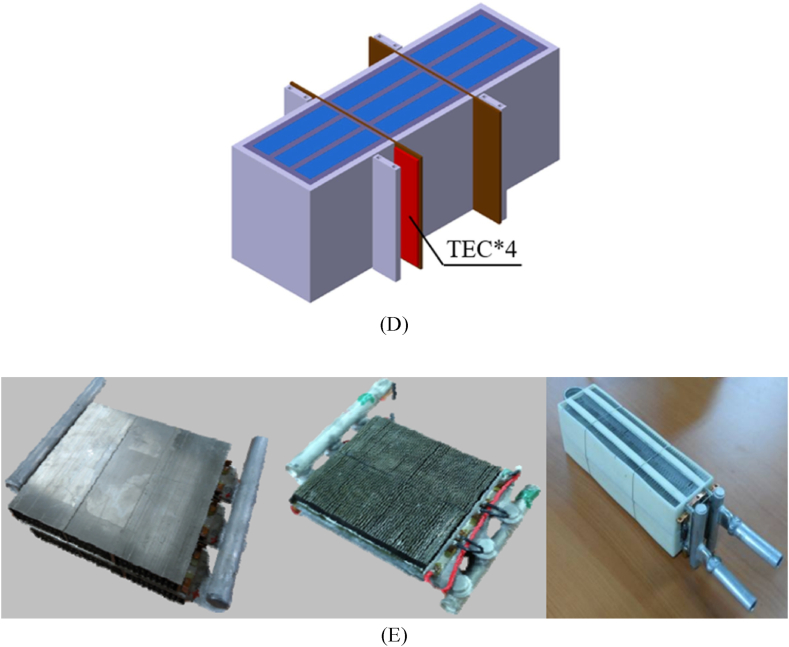
(A) Configuration of the battery and thermoelectric system, showcasing variable fin shapes [116] (B) Battery cooling based on TEC with variable fin arrangement orientations [96] (C) Fin framework of a TEC based PCM Li ion BTMS with varying fin length and thickness [117] (D) The fin-based three-dimensional model of BTMS [88] (E) Engineered Proto TEM battery cooling modules (Proto 1, Proto 2, and Proto 3, respectively) [50]

The thermoelectric-based BTMS incorporates a thermoelectric device, where the cold side is affixed to the battery surface, and the hot side is connected to the fins of a heatsink. Sait [116] conducted a simulation to model the cooling mechanism of a plate-type LIB cell by using thermoelectric principles as depicted in Fig. 4 (A). The thermoelectric device is configured so that the cold side is positioned in contact with the battery. In contrast, the hot side is affixed with a heatsink including several pin fins. Various forms of pin fins, including circular, elliptical, triangular, as well as trapezoidal, were used in an experimental setup where a channel was filled with alumina nanofluids (NFs) in a saturated state. The study's findings revealed that the use of triangular pin-fins led to the lowest temperature seen on both the heatsink and the battery. Conversely, the employment of elliptical pin-fins resulted in the highest temperature recorded on both the battery and the heatsink. Liu et al. [96] developed a hybrid BTMS by merging TEC and PCM. By undergoing phase shift and heat absorption, the PCM stores heat from batteries. By means of the fins, the TECs remove surplus heat from the PCM in the manner of a heat pump. The influence of fin arrangement on TEC performance was investigated in this research. According to the findings (see Fig. 4 (B)), the fins should be positioned normal to battery direction, which has better thermal conductivity, so that batteries' heat can be transferred to the fins more rapidly and efficiently, boosting the TEC's performance. This variant has shorter fins along the width of the battery, making it more compact and lightweight for BTMS. To ensure the optimal operational temperature of batteries, Luo et al. [117] created a unique BTMS in conjunction with PCMs and TECs, where a fin framework (shown in Fig. 4 (C)) was utilized to promote the heat transmission. The results showed that increasing the TEC input current, fin thickness, and fin length may lower the PCM liquid percentage and maximum temperature. Nevertheless, TEC input current and fin thickness have very little effect on temperature differential, even though increasing fin length may reduce it. The optimal thickness and length of the fins, which are determined to be 3 mm and 7 mm, respectively, through numerical analysis.

Liu et al. [88] introduced an active-passive BTMS which incorporates fins to optimise the integration of PCM and TECs, as illustrated in Fig. 4 (D). The objective was to prolong the operational duration of the battery by maintaining its temperature within 328.15 K during rapid discharge, hence improving the temperature consistency during extended periods of usage. The study focused on analyzing the impact of varying fin thicknesses on thermal performance. At varying discharge rates, the optimal fin thickness was then compared to the BTMS without fins. The findings indicated that, in comparison to the non-fins model, the 4 mm-fins model demonstrated superior cooling capacity and more efficient cooling performance of the TEC at variable discharge rates of the battery. At a 2C discharge rate, the 4 mm-fins model exhibited a temperature control time that was 52.5 % greater than the model devoid of fins. At a 1.5C discharge rate, the battery's maximum temperature was effectively regulated at 314.46 K by the 4 mm-fins model. Still, the no-fins model saw a continuous increase in maximum temperature. The temperature uniformity was superior in the 4 mm-fins model, based on the comparative findings of temperature difference at various discharge rates of the battery. In research by Kim et al. [50], three distinct kinds of 1 kW thermoelectric battery cooling modules were constructed with various fins for the air supply side, as depicted in Fig. 4 (E). The initial prototype (Proto 1) weighed 2.547 kg overall and had a TEC module in conjunction with dimple fins and a three-row water tube route. The dimple fins were constructed from 25 mm H x 178 mm W aluminium plate-style tubes. To construct the water-cooling jacket for Proto 1, welded water tubes were utilized. In Proto 2, water tubes of the welded kind were used, with aluminium louver fins chosen. These fins had dimensions of 82.4 mm width and 16 mm height, with a thickness of 0.16 mm. Subsequently, the total weight had decreased to 1.862 kg. As opposed to the welded water tubes, extrusion tubing was utilized in Proto 3. Louvre vents made of thin aluminium were chosen for air-side thermal exchange in this prototype. The dimensions of the fins were a diameter of 58 mm and a height of 10.1 mm. The thickness of the fin was 0.09 mm. Comprising the connecting pipelines and wiring, the total weight was significantly diminished to 747g. Additionally, a 1-row water conduit was utilized to reduce the weight of Proto 3. The results demonstrated that the air-side heat transfer fins had a significant impact of on the 1 kW TEC's overall COP.

Subsequent to these discoveries, it is evident that the design and configuration of condenser fins have an impact on the efficacy of TECs. The thermal efficacy of the system is influenced by the configuration of the fins. Triangular fins provide superior cooling due to their larger surface area and sharpened edges, whereas elliptical fins perform inadequately due to their convex structure. Circular and trapezoidal fins provide intermediate performance by achieving a balance between structural simplicity and surface area. The thermal efficacy of the system is also influenced by the thickness of the fins. In general, fins that are thicker have a higher thermal conductivity, which facilitates the transfer of heat more efficiently. If the fins are excessively dense, the system's weight may increase without a substantial improvement in cooling efficacy. Additionally, fin arrangements are essential for optimizing heat dissipation. Thermal conductivity is enhanced by aligning fins perpendicular to the battery surface, while shortened fins facilitate system compactness. The TEC's efficacy is optimised by ensuring uniform cooling through the use of appropriate fin spacing and orientation.

## 13 Control of TEC based Li- ion BTMS

Some approaches are commonly utilized for controlling thermal management systems, such as proportional integral derivative (PID) [118], fuzzy control [119], and finite-state machine methods [120]. However, the generation of rules in these approaches relies on past data. Control outcomes will only be adequate if the experience is sufficient.

### 13.1 PID control

PID control is a conventional and extensively employed approach to temperature regulation in TEC-based BTMS. The TEC's input current is adjusted by this control strategy in response to the temperature error, which is the discrepancy between the desired and actual battery temperatures. To rectify this error, the PID controller implements derivative, integral, and proportional terms. The TEC input is adjusted by the proportional term in response to the current error, the integral term addresses accumulated errors over time, and the derivative term predicts future errors based on the rate of change. Under steady-state conditions, PID control is effective in preserving a consistent temperature. Nevertheless, it may encounter difficulties due to the non-linear behaviour of TEC systems, particularly during rapid temperature fluctuations or variable load conditions. This could result in potential issues such as sluggish response times or the necessity for frequent recalibration [[121], [122], [123]].

### 13.2 Fuzzy logic control

Fuzzy logic control offers a more adaptable and flexible method for regulating TEC systems, especially in the context of non-linearities and uncertainties. In contrast to PID control, which depends on accurate mathematical models, fuzzy logic control employs a rule-based methodology to manage imprecise and ambiguous data. Fuzzy logic modifies the TEC's cooling rate by using a series of fuzzy rules and membership functions in response to temperature variations and other input parameters. This facilitates more seamless transitions and enhanced adaptability in intricate systems. While fuzzy logic control effectively addresses non-linearities and offers superior temperature management relative to conventional approaches, the design of a fuzzy control system necessitates the development of exact rule sets and membership functions, a process that may be intricate and demands specialised knowledge [31,122,123].

### 13.3 Machine learning-based approaches

Machine learning methodologies are a state-of-the-art solution for enhancing TEC efficiency in BTMS. These technologies use data-driven models to forecast the thermal performance of the battery and modify the TEC operation in real-time. Machine learning techniques, including neural networks, analyze historical and operational data to predict temperature trends and enhance cooling tactics. This method allows highly adaptable control, enhancing temperature regulation and energy economy. Nevertheless, machine learning-based controllers need considerable computer resources and high-quality data for successful training [31,121].

Recent breakthroughs have investigated the integration of machine learning approaches with Nonlinear Model Predictive Control (NMPC). This hybrid methodology combines machine learning to forecast the battery's forthcoming temperature trajectory and nonlinear model predictive control to optimise the thermoelectric cooler's input for sustaining desirable operational conditions. Furthermore, the use of evolutionary algorithms with NMPC improves the system's capacity to optimise energy economy and cooling effectiveness, hence providing reliable performance amongst fluctuating external factors like as traffic, ambient temperature, and driving behaviour [121,122]. Although PID and fuzzy logic controllers are characterised by their simplicity and flexibility, machine learning-based methodologies provide superior accuracy and efficiency in optimizing TEC-based battery thermal management systems. Each technique has distinct advantages and drawbacks, and the selection of a control strategy is contingent upon the unique needs and circumstances of the TEC system in lithium-ion battery thermal management.

Furthermore, these solutions disregard the problem of energy optimization. Some BTM solutions use nonlinear model predictive control [124] and back-stepping control [125] to cope with the thermal management system's nonlinearity, various constraints, and energy optimization. Although these approaches provide a greater control effect, the performance and efficiency of the BTMS remain restricted because of the following two factors: First, manufacturing variations across batteries cause their thermoelectric characteristics to vary, even within the same pack. Furthermore, the model's accuracy will be affected by extraneous factors such electromagnetic noise, inaccurate measurements, weather conditions, heavy traffic, and driving behaviour. All of these elements will have a significant impact on BTMS accuracy. In order to manage the uncertainty of the system, a comprehensive model predictive control strategy is necessary, according to the reasoning above.

**Fig. 5:**
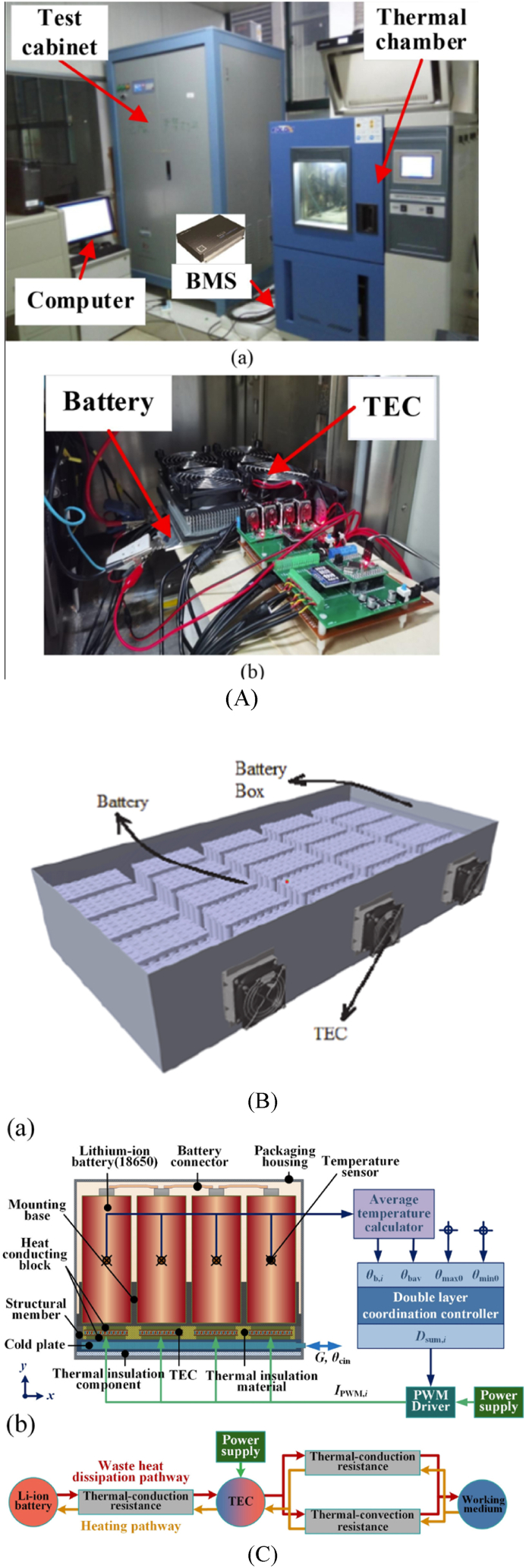
(A) Platform for experimentation. (a) BTS: battery test system; (b) Temperature control test platform [121] (B) Simulation studies of controllers for BTMS with multi-thermoelectric modules [127] (C) Conceptualization of thermal management using TEC technology to achieve great temperature uniformity [128].

Using the Fuzzy PID algorithm, Liu et al. [126] developed a rapid temperature control thermal management system for automobile batteries. Using a Fuzzy PID algorithm, the temperature of the battery cell is rapidly regulated by varying the magnitude and direction of the TE device current in a timely manner. Cui et al. [121] introduced a resilient predictive BTMS approach with TEC technology, as shown in Fig. 5 (A). Ensuring that the LIB remains within the optimal operating temperature range is the objective, while also minimizing energy use. Initially, a thermal resistance-based heat transfer model of TEC was developed, considering the impact of both the heat sink and fan on the modelling outcome. Subsequently, a distributed battery thermal model was created employing the finite difference approach. This modelling approach takes into consideration the impact of tab heat-generating characteristics on the distribution of temperature, resulting in improved model accuracy. Subsequently, a model for managing the thermal conditions of the battery was created, and a resilient Nonlinear Model Predictive Control (NMPC) approach relying on Neural Networks (NN) was suggested to regulate the battery's temperature. This approach could attain superior control precision in the presence of disturbances. The results of further stability research demonstrated the convergence of the suggested observer. Nasir et al. [127] investigated a modified lithium-ion battery thermal management system through simulation-based investigations (see Fig. 5 (B)) employing PID and Null-Space-based Behavioural (NSB) controllers. This endeavour aimed to maintain the optimal temperature for battery life while consuming minimal power. Thermoelectric modules were employed in conjunction with collaborating controllers to reduce the amount of electricity consumed during the refrigeration procedure. In contrast to the PID controller, the NSB controller demonstrated a 20 % reduction in power consumption, expedited temperature restoration to the set point, and improved temperature uniformity across the battery cells. Chang et al. [128] developed a fuzzy-PID dual-layer coordinated control approach that utilizes TECs to address the thermal issues of the LIB pack used in space applications, as depicted in Fig. 5 (C). The technique aims to achieve high-temperature uniformity. A model was developed to analyze the behaviour of the BTMS. The study focused on the open-loop characteristics of the LIB pack during an orbital cycle. Factors such as discharge time, the satellite surface's thermal-optical properties, and the working medium's volume flow rate in the cold plate were considered. Numerical analysis was conducted to understand the system's performance during orbital operation. The efficacy of the fuzzy-PID dual-layer coordinated controller is assessed through numerical evaluation. The findings demonstrate its ability to sustain the average temperature of the LIB pack within the 15–35 °C temperature range that is desirable, even in the presence of intricate thermal disruptions. The maximum temperature deviation is limited to 1.7 °C, which is 76.7 % lower than the absence of active control. Additionally, the minimum temperature deviation is a mere 0.06 °C.

## 14 Limitations and Future direction of TEC based BTMS

Similar to other systems, TEC may also possess certain drawbacks, which include.

• Material costs: The expense of materials is a significant obstacle in making TEC widely accessible at an affordable price [129,130]. The pricing of frequently used Bi_3_Te_2_ or any Bi3Te2 alloy is much higher compared to other accessible thermoelectric (TE) materials. For instance, Mg_2_Si_0.6_Sn_0.4_ is priced at $4.04/kg, but Bi_3_Te_2_ is priced at $125/kg [129].
• The Widemann-Franz law states that the product of electrical resistivity and thermal conductivity stays constant for a thermoelectric material under fixed temperature conditions. This observation indicates that there are disadvantages to enhancing the figure of merit (FOM) of thermoelectric materials [131]. Lately, several researchers have been endeavoring to enhance the Figure of Merit (FOM) by intentionally changing the crystal structure of the material [[132], [133], [134]].
• TEC systems suffer from a fundamental limitation in terms of their inherent poor efficiency [135]. Typically, TEC provides an efficiency ranging from 10 to 15 % of the theoretical Carnot cycle refrigerator. Conventional compression cycle systems, namely reverse Rankine systems that use compression/expansion, often attain an efficiency range of 40–60 % [45,136]. This efficiency should be contrasted with the efficiency of the system in question. TEC is often used in situations when the benefits of its solid-state nature are more significant than its inferior efficiency. TECs have a high degree of sensitivity to changes in input power. If the design is not well-executed, the efficiency of the system decreases significantly [135].
• The figure of merit (Z) is used to assess the performance of the TE materials/legs, with a low value indicating poor performance [137]. Typically, the Z parameter is represented as ZT, which is a dimensionless quantity defined as $\text{ZT}=\frac{\alpha^{2}}{\rho \lambda }T$, where α represents the Seebeck effect, ρ represents the electrical resistivity, λ represents the thermal conductivity, and T represents the temperature [138]. TECs often have lower performance (COP less than 2.4) compared to conventional refrigeration systems (COP more than 3) owing to their low ZT value [139,140]. ZT is a dimensionless figure of merit that is an important factor in determining the efficiency of thermoelectric materials [141,142].
• In comparison to others, the operating lifespan is quite short (1–3 years) [50,143].

### 14.1 Future direction

**Fig. 6:**
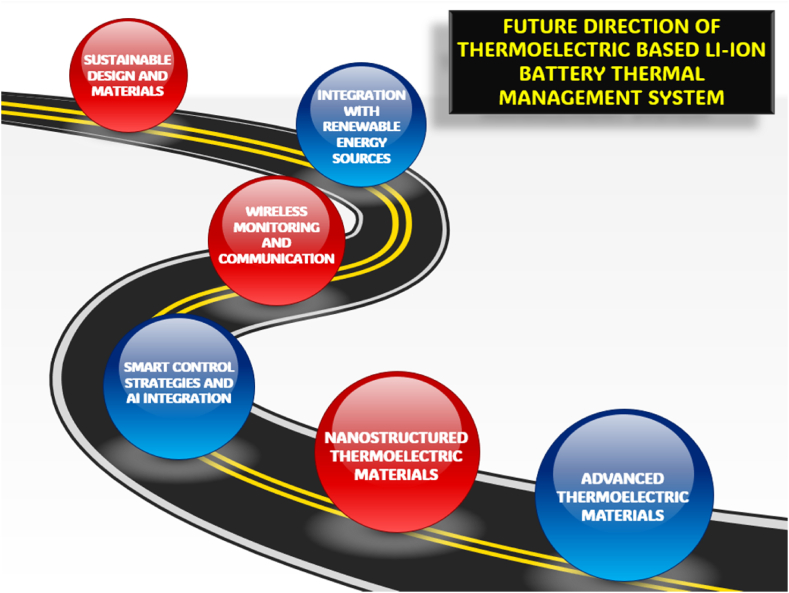
Future direction of thermoelectric based Li-ion BTMS.

Given the increasing need for energy-efficient and sustainable technologies, it is imperative to explore enhanced thermal management methods for LIBs. LIBs are extensively used in many applications, spanning from electric automobiles to portable devices. Nevertheless, they encounter obstacles with the production of heat when undergoing the processes of charging and discharging, which might have an effect on their efficiency, safety, and longevity. TEC is a potential method for actively controlling the temperature of LIBs. Thermoelectric modules use the Peltier effect to transfer heat from the battery for maintaining the ideal working temperatures. In order to advance this technology, it is crucial to contemplate future trajectories that include advancements in materials, control techniques, and sustainability (depicted in Fig. 6). Some of the prospective directions to be considered are.

• **Advanced Thermoelectric Materials:** The pursuit of improved thermoelectric materials entails the identification of compounds exhibiting exceptional thermoelectric characteristics [144]. The superior performance of materials depends on a meticulous balance of hierarchical trade-offs, such as the interaction between structural organisation and randomness, stability and instability of phases, convergence and splitting of energy bands, effective mass and mobility, covalency of bonds, and ionicity. Currently, the topic of TE materials research is at the forefront of scientific advancement due to the exploration of several ideas such as topological states, the Rashba effect, the spin Seebeck effect, resonant bonding, resonant levels, and anharmonicity [145]. Materials with high Seebeck coefficients and low thermal conductivity may greatly improve the effectiveness of the thermoelectric cooling process, resulting in more efficient temperature control for Li-ion batteries.
• **Nanostructured Thermoelectric Materials:** Introducing nanostructures into thermoelectric materials at the nanoscale results in distinct features that may improve the transportation of electrons and phonons. Nanoscale or nanostructured morphologies may be used to achieve ZT enhancement [134,146,147]. Nano-structuring may amplify the density of states (DOS) near the Fermi level by quantum confinement, resulting in an elevation of thermopower. This phenomenon offers a means to separate thermopower from electrical conductivity. The objective of this technique is to enhance thermoelectric efficiency by enhancing the material's capacity to convert heat into electrical energy and vice versa [148].
• **Smart Control Strategies and AI Integration:** By incorporating artificial intelligence (AI) and using intelligent control algorithms, thermal management systems may achieve adaptive and dynamic regulation. AI algorithms can analyze live data from temperature sensors, accurately forecast and react to fluctuating circumstances to enhance the efficiency of the thermoelectric cooling system [149].
• **Wireless Monitoring and Communication:** The integration of wireless monitoring and communication capabilities improves the accessibility and remote control of thermoelectric-cooled LIBs. This technology facilitates instantaneous data interchange, so allowing effective surveillance and regulation, even in distant or demanding settings.
• **Integration with Renewable Energy Sources:** The integration of thermoelectric-cooled LIBs with renewable energy sources results in the development of self-sustaining energy solutions. The system may function autonomously by integrating thermal management with renewable energy, supplying electricity for cooling and other purposes in an eco-friendly way [150,151].
• **Sustainable Design and Materials:** Highlighting sustainability in thermal management systems entails investigating environmentally friendly materials and design methodologies. This strategy seeks to minimize the ecological footprint of battery technology, in line with worldwide endeavors for more environmentally friendly and enduring energy solutions [92,152]. Scientists are anticipated to seek additional deviations from the existing material selection criteria in the coming years, such as the creation of additional high-performance TE materials composed of elements that are abundant on Earth and non-toxic [145].

## 15 Conclusion

In conclusion, this thorough analysis has explored the complex field of Thermoelectric Technologies for Improved Thermal Management in LIB Systems. The review has thoroughly examined the advancements and gaps in this growing subject by extensively exploring many aspects, including the fundamental working principles, experimental setups, and investigations. An examination of TEC-based hybrid BTMS cooling methods has unveiled a variety of approaches, such as heat pipe-cooled thermoelectric BTMS, air-cooled thermoelectric BTMS, and PCM-cooled thermoelectric BTMS. Every approach has undergone a rigorous evaluation, illuminating its merits and limitations. Extensive research has been conducted on the effects of heat sink fin shape, thickness, and arrangement in TEC, yielding valuable insights into optimizing these parameters to achieve improved performance. Furthermore, the discussion on the Control of TEC-based Li-ion BTMS has highlighted the need for effective management and control in attaining optimum thermal results. Recognizing the limits of TEC-based BTMS, this review presents a clear summary of the difficulties encountered, urging researchers to overcome these obstacles in future endeavors. The Future Directions section provides an overview of attractive areas for research and development, emphasizing the potential for innovation and significant advancements in addressing current limits. The ongoing advancement of thermoelectric technology has the potential to profoundly transform the field of thermal control in LIBs, with wide-ranging ramifications that extend beyond the scope of this review.

## CRediT authorship contribution statement

**Mehwish Khan Mahek:** Writing – original draft. **Mohamad Ramadan:** Writing – review & editing, Supervision. **Sharul Sham bin Dol:** Writing – review & editing. **Mohammed Ghazal:** Writing – review & editing. **Mohammad Alkhedher:** Writing – review & editing.

## Declaration of competing interest

The authors declare that they have no known competing financial interests or personal relationships that could have appeared to influence the work reported in this paper.
